# Effects of Prenatal Exposure to Bisphenol A Substitutes, Bisphenol S and Bisphenol F, on Offspring’s Health: Evidence from Epidemiological and Experimental Studies

**DOI:** 10.3390/biom13111616

**Published:** 2023-11-05

**Authors:** Raya Algonaiman, Abdulkarim S. Almutairi, Muath M. Al Zhrani, Hassan Barakat

**Affiliations:** 1 Department of Food Science and Human Nutrition, College of Agriculture and Veterinary Medicine, Qassim University, Buraydah 51452, Saudi Arabia; 411200162@qu.edu.sa; 2 Al-Rass General Hospital, Qassim Health Cluster, Ministry of Health, Ibn Sina Street, King Khalid District, Al-Rass 58883, Saudi Arabia; abdulkarimsa@moh.gove.sa; 3 Department of Applied Medical Science, Applied College, Bishah University, Bishah 67616, Saudi Arabia; muath@ub.edu.sa; 4 Department of Food Technology, Faculty of Agriculture, Benha University, Moshtohor 13736, Egypt

**Keywords:** bisphenols, bisphenol S, bisphenol F, public health, nutrition, newborn, pregnancy

## Abstract

Pregnancy and lactation are critical periods for human well-being and are sensitive windows for pollutant exposure. Bisphenol A (BPA) is well demonstrated as a toxicant and has been replaced in the plastic industry with other bisphenol analogs that share similarities in structure and characteristics, most commonly Bisphenol S (BPS) and Bisphenol F (BPF). Maternal exposure to BPS or BPF can result in their accumulation in the fetal compartment, leading to chronic exposure and potentially limiting normal fetal growth and development. This review summarizes considerable findings of epidemiological or experimental studies reporting associations between BPS or BPF and impaired fetal growth and development. Briefly, the available findings indicate that exposure to the two bisphenol analogs during pregnancy and lactation can result in multiple disturbances in the offspring, including fetal growth restrictions, neurological dysfunctions, and metabolic disorders with the potential to persist throughout childhood. The occurrence of premature births may also be attributed to exposure to the two bisphenols. The possible mechanisms of actions by which the two bisphenols can induce such effects can be attributed to a complex of interactions between the physiological mechanisms, including impaired placental functioning and development, dysregulation of gene expression, altered hormonal balance, and disturbances in immune responses as well as induced inflammations and oxidative stress. In conclusion, the available evidence suggests that BPS and BPF have a toxic potential in a compartment level to BPA. Future research is needed to provide more intensive information; long-term studies and epidemiological research, including a wide scale of populations with different settings, are recommended. Public awareness regarding the safety of BPA-free products should also be enhanced, with particular emphasis on educating individuals responsible for the well-being of children.

## 1. Introduction

Bisphenols are a group of synthetic chemicals widely used in producing plastics and resins, found in various types of frequently used products, such as food and beverage containers, plastic bottles, baby formula bottles, thermal paper receipts, and dental sealants. Bisphenol A (BPA), the most used in the plastic industry, has raised concerns due to its potential toxicity as an endocrine disruptor, meaning that it can mimic estrogen and disrupt the hormonal balance. Studies have confirmed the link between BPA exposure and the incidence of various health issues, including hypertension, obesity, type 2 diabetes, cardiovascular diseases, and cancer [1,2,3]. As a result, considerable efforts have been established since the late 2000s to limit BPA exposure; many countries and regulatory agencies have implemented restrictions on its use to ensure safer alternatives in consumer products, especially those intended for infants and children [4]. However, despite their structural and characteristic similarities, other bisphenol analogs like bisphenol S (BPS), bisphenol F (BPF), and bisphenol AF (BPAF) have replaced BPA. These analogs become extensively available in a wide range of products regarded as safe and labeled “BPA Free”, leading to a remarkable increase in their exposure. Some general populations showed a decreasing pattern in BPA urinary concentrations between 2000 and 2014, while BPS and BPF concentrations constantly increased [5].

Bisphenol analogs have been detected in breast milk, amniotic fluid, and fetal tissues, indicating that their exposure can occur during critical developmental periods in early life, which represent extensive sensitivity to environmental pollutants [6]. Furthermore, despite the low maternal–fetal placental transfer and the low exposure dose, repeated maternal exposure to BPA alternatives, primarily BPS, can result in the accumulation of these substances in the fetal compartment, leading to chronic exposure [7]. BPA alternatives also exhibit a significantly longer half-life in the fetal compartment compared to the adult circulation, with a 20-fold difference reported [8]. Indeed, the mean concentrations of BPS in urinary samples of 134 newborns were found to be 0.09 μg L^−1^, while their mothers had a concentration of 0.01 μg L^−1^ [9]. Therefore, the safety of BPA analogs or alternatives, particularly during early life development, should raise concerns. Indeed, recent studies suggested that certain BPA alternatives may exert similar endocrine-disrupting effects, indicating their potential to promote similar health issues. Some studies have also reported associations between BPS and BPF exposure, the most used substitutes for BPA, and certain health issues among adult individuals, including hypertension, cognitive dysfunctions, obesity, type 2 diabetes, cardiovascular diseases, and cancer [10,11,12,13,14]. In this review, the current existing research in relation to the potential toxicity of BPS and BPF exposure during fetal growth and development will be discussed.

## 2. Search Strategy

For this narrative review, eligible research articles were searched on the relevant databases, including PumbMed, ScienceDirect, and EBSCO, with a further search on Google Scholar, encompassing articles published up to August 2023. The search was conducted using a combination of keywords, including bisphenol A, bisphenol S, bisphenol F, prenatal exposure, maternal exposure, exposure assessment, pregnancy, fetus, newborns, and offspring. The included research articles consisted of epidemiological studies (cohorts and case studies) and experimental studies conducted on animal models. To maintain consistency and facilitate effective understanding, research studies written in languages other than English were excluded.

For the selection process of the epidemiological studies, four inclusion criteria were employed: (i) utilization of maternal urinary or serum samples for assessing prenatal exposure, (ii) assessments conducted during at least one trimester, (iii) assessment of a specific risk factor on the fetus or offspring health, and (iv) expressing the exposure level as µg/L, ng/mL, or nmol/L of the urinary/serum samples, with the exclusion of studies that adjusted urinary values to urinary creatinine in order to facilitate and enable a more streamlined comparison of the data. Among the pool of 31 population studies assessed, a total of 15 studies (consisting of 1 case–control study and 14 cohort studies) met the predefined selection criteria for eligibility. Further, to ensure uniformity, all exposure values were adjusted and presented as µg/L. This adjustment allowed for consistent and standardized reporting of the exposure levels across the selected studies. It was necessary to facilitate accurate comparisons and analysis of the data, promoting reliable findings and conclusions.

For the selection process of the experimental studies, two inclusion criteria were employed: (i) intentional administration of the specific bisphenol compound (BPS, BPF, or a mixture of both) to the model dams either before pregnancy, during pregnancy, during lactation or a combination of these periods, and (ii) assessment of a specific risk factor on offspring of the maternal models. By employing these inclusion criteria, the selection process aimed to identify experimental studies that examined the subsequent impact of maternal exposure to the selected bisphenol on a particular risk factor in their offspring. Out of a total of 42 animal studies examined, 17 studies were found to meet the established eligibility criteria.

## 3. Effects of BPS or BPF Prenatal Exposure on Offspring’s Health Outcomes

The possibility of prenatal exposure to BPS or BPF, as the most commonly used BPA substitutes, affecting the offspring’s health is discussed in depth in this section. Based on the available findings observed in epidemiological and experimental studies that relied on either maternal urinary concentrations of bisphenols, which are commonly used as reliable indicators of exposure, or on direct exposure via different routes of administration on animal/tissue models as presented in Table 1 and Table 2. Further explanations on the possible mechanisms of action are also presented concerning each certain effect.

### 3.1. Effects on Fetal Growth

In multiple epidemiological studies, the association between maternal urinary concentrations of BPS or BPF during pregnancy and birth anthropometric parameters was investigated; in a cohort study, a significant decrease in birth length by 0.12 cm with an increase in the ponderal index by 0.04 g/cm^3^ × 100 was associated with higher maternal urinary concentrations of BPF. The shorter birth length was notably more pronounced in female newborns compared to males, indicating the potential influence of fetal sex in changing the vulnerability to bisphenols *in utero* exposure [15]. Similar sex-specific results were also observed in another cohort [19]; maternal BPF urinary concentrations were associated with decreased birth weight and ponderal index in male newborns but not females. In other interesting results, Yang et al. [15] found an inverted U-shaped relationship between maternal BPF urinary concentrations and decreased birth length, meaning that the adverse effects on birth length were observed only at higher and lower concentrations. In contrast, average concentrations showed no significant impact. Such results can be explained by the varying roles of BPA at different doses; lower doses of BPA can mimic estrogen and stimulate cellular responses, while higher doses were shown to promote the binding to estrogen receptors [48]. Therefore, BPA alternatives may also exhibit similar roles due to their structural similarities.

Meanwhile, the two cohorts, J. Liang et al. [19] and Yang et al. [15], observed such adverse associations with BPF exposure but not BPS, which might indicate that BPS is a safer alternative compared to BPF. However, Yang and his colleagues only measured the third trimester’s urinary samples, leading to cross-sectional observational data. Different periods of gestation may show different susceptibility to bisphenols’ adverse effects on fetal growth; an earlier cohort by Hu et al. [16] reported that higher maternal urinary concentrations of both BPF and BPS during different trimesters, mainly first and second, had a significant linear association with decreased birth length, birth weight, and ponderal index. Interestingly, BPA exposure in this cohort study showed null associations, which may indicate that BPA alternatives are more potent on fetal growth. However, another cohort reported significant changes in birth head circumference associated with maternal urinary concentrations of BPA but not its alternatives (BPF or BPS). However, it should be noted that this cohort was also limited to the assessment of only one trimester, as well as relying on a single spot of urine sample, which might result in misclassification of exposure assessment due to the short half-life and rapid metabolism of BPs in the human body [17]. In contrast, another study measured maternal urinary concentrations of BPs across all trimesters and found significant associations between average BPS concentrations throughout pregnancy and increased fetal head circumference. Significant trimester-specific associations were also observed; any detection of BPS during the first trimester was associated with larger fetal head circumference and fetal weight in both the second and third trimesters [18]. Such significant association was only observed with BPS exposure but not with other BPs; it has been indicated that BPS exposure during pregnancy could affect maternal hormone levels, leading to changes in fetal growth depending on the exposure period [49,50]. In another study, serum samples were used to assess the exposure, and it found that BPF but not BPS was significantly associated with lower birth weight and ponderal index [19]. Another study found that both BPF and BPS during some trimesters had significant associations with lower birth weight, birth length, and ponderal index. Another study also reports consistent significant results with both BPS and BPF but in opposite directions; BPS exposure decreased birth weight while BPF increased. However, the mixture of the two BPs generally resulted in decreased birth weight. Suggesting that solo chemical exposure and mixture exposure might show different adverse effects on the same health outcome [20]. Furthermore, in a twin birth cohort, consistent associations were also observed with both BPF and BPS; the second trimester’s maternal urinary concentrations of the two BPs were associated with increased birth weight. However, the significant association was only observed with BPF exposure but not BPS [21].

The possible mechanism of action in which BPA alternatives can promote changes in fetal growth parameters might be related to impairments in skeletal muscle development. In pregnant sheep exposed to BPS during gestation, their fetuses showed larger myofibers (muscle fiber hypertrophy) attributed to an increase in the expression of myogenic regulatory factors (MRFs), with females showing more potent results than males [30]. Larger fetus myofibers can result in larger muscle mass, potentially leading to a higher birth weight. However, birth size is not directly determined via the MRFs; it is rather influenced by other multiple significant factors, including maternal health and placental development. Indeed, abnormalities in placental development can play significant roles in fetal growth; in placental tissues of mice dams exposed to BPS or BPA before pregnancy, results showed a decrease in the placental content of serotonin [31]. In addition to serotonin’s key roles in neurodevelopment, alterations in placental serotonin levels could impact fetal growth outcomes due to its role in inducing autocrine/paracrine effects, which play significant roles in fetal growth and development. Indeed, deficiencies in placental serotonin were indicated in several studies as a sign of fetal growth restriction [51,52,53].

Furthermore, BPS exposure can lead to other placental deficiencies; in BPS-exposed placentas of pregnant sheep [32], results showed a decrease of 50% in the expression of E-cadherin protein, which is a cell adhesion protein that plays a crucial role in maintaining the integrity and function of the placental barrier. Decreased E-cadherin protein levels can disrupt normal placental development and function, impairing fetal growth [54,55]. The BPS-exposed placentas also showed a decrease of 20% in the binucleate cells [32], which are specialized cells found in the placenta and play key roles in placental structure and function; therefore, their reduction can indirectly lead to impairments in fetal growth as a result of impaired placental functioning [56,57,58]. In other words, inadequate placental function can limit fetal nutrient delivery, leading to fetal growth restrictions [56]. Gingrich et al. [32] also indicated that the observed impairments in the BPS-exposed placental were much more potent compared to those exposed to BPA, suggesting that BPA alternative might promote more toxic effects on early development than BPA.

In brief conclusion, prenatal exposure to BPA alternatives may promote toxic effects on fetal growth outcomes in a similar manner or even worse than BPA. The evidence from the epidemiological studies showed that higher maternal urinary BPS or BPF concentrations can result in alterations in fetal growth, leading to changes in birth size, including birth weight, birth length, head, and ponderal index. Furthermore, trimester- and sex-specific associations were observed, indicating that the potency of BPA alternative exposure may depend on both the sensitivity of the exposure period and the sex of the fetus. The possible mechanism of action beyond BPA alternatives in promoting such impairment in fetal growth outcomes might be linked to disturbances in placental function and development, as evident by a couple of experimental studies on pregnant animal models. BPS-exposed placentas showed significant reductions in multiple key parameters related directly or indirectly to fetal growth and development. Further research is needed to confirm the possible toxicity of BPA alternatives on fetal growth, and further in-depth research is suggested to uncover the related possible mechanisms fully.

### 3.2. Effects on Gestational Period and Preterm Births

The *in utero* exposure to BPA alternatives was linked in multiple studies to a short gestational period leading to preterm births; in a case–control study, maternal BPS urinary concentrations during the last trimester were significantly associated with increased odds of preterm birth, including spontaneous and placental [22]. Similarly, BPS maternal urinary concentrations that were detected in nearly 48% of samples showed a significant linear dose-dependent relationship with shorter gestational weeks [15]. However, another study reported that only BPA, but not BPS, had a significant association with decreased gestational period and increased preterm births. Although the detection rate of BPA reached nearly 68% of the samples, this cohort was limited to a smaller sample size, not exceeding 1000 in comparison to the latter, which included 1197 mother–newborn pairs [23]. A larger sample size enhances the statistical power and highlights the ability to generalize the findings. Indeed, in another cohort that included 2023 mother–infant pairs, the mixture of BPs (BPS, BPF, and BPA) was shown to significantly relate to increased preterm births, with BPA sharing the least contribution (26.8%). At the same time, BPS and BPS had a contribution of 43.7% and 29.6%, respectively [24].

Consistently, in pregnant rat models exposed to BPF during pregnancy, over 80% of the dams exhibited spontaneous abortions [33]. Such a high rate was attributed to decreased corpora lutea, which are temporary endocrine structures that develop in the ovaries after ovulation and play a vital role in early pregnancy by producing high levels of hormones, primarily progesterone. Adequate progesterone is crucial for maintaining a stable pregnancy by suppressing labor and promoting uterine muscle relaxation. Therefore, insufficient progesterone due to decreased corpora lutea can increase the risk of preterm birth [59,60,61,62]. Insufficient progesterone levels during pregnancy can also be attributed to elevated estrogen levels, resulting in an imbalance between progesterone and estrogen. This progesterone/estrogen ratio imbalance is considered a potential mechanism by which BPA and its alternatives contribute to preterm birth. BPA and its alternatives are well-demonstrated as endocrine disruptors, meaning they could mimic or interfere with estrogen, leading to imbalanced hormonal levels. While progesterone promotes a stable pregnancy, estrogen is crucial in inducing labor by promoting significant increases in myometrial contractions [63,64,65].

Furthermore, exposure to BPA and its alternatives could lead to preterm birth by promoting impairments in the decidua, placenta, and fetal membranes that may counteract uterine muscle relaxation and promote fetal membrane rupture, resulting in preterm birth [66,67]. BPA and its alternative can also stimulate placental cell apoptosis, leading to abnormal placental development and potentially leading to premature babies [22,23]. In addition, maternal exposure to BPA or BPS was linked to a decrease in placental weight, which can also lead to preterm birth [68]. Disturbances in the placenta–brain axis were also reported, which can lead to abnormal placental responses and potentially result in early pregnancy loss and placental diseases such as preeclampsia [31].

To state a conclusion, there is a piece of evidence suggesting the possible link between maternal exposure to BPA alternatives and the incidence of preterm birth. In some studies, BPA alternatives were shown to promote more potent effects on the stability of pregnancy compared to BPA, suggesting that BPA alternatives can not be considered safe. These chemicals can promote preterm birth via multiple possible mechanisms, including stimulating progesterone/estrogen imbalance, promoting alterations in the normal placental functioning, decreasing placental size, and disrupting the placenta–brain axis. On the other hand, some of the reported findings were only observed among women carrying female fetuses [15], while others were only with male fetuses [24], indicating that the vulnerability to BPs *in utero* exposure might strongly depend on the fetus’s sex. Further investigations are recommended to uncover the possible mechanisms underlying the sex-specific results comprehensibly.

### 3.3. Effects on Cognitive Functions and Psychomotor Skills

*In utero* exposure to BPA alternatives may also promote adverse effects on the children’s cognitive functions in the long term; in a cohort study of mother–child pairs [25], BPF *in utero* exposure was significantly associated with decreased children’s IQ (Intelligence Quotient) at 7 years old. With each log-unit increase in BPF concentrations, IQ scores decreased by ~2 points. Such significant effects on cognitive functions were more pronounced in boys than in girls. Similarly, another cohort showed a significant association between BPS prenatal exposure and decreased psychomotor development index by 7.6 points observed among boys but not girls [26]. It should be noted that there was no significant difference in the proportion of boys and girls in these cohorts, with approximately an equal distribution of around 50% for both genders. Providing more evidence of the ability of sex differences to affect the vulnerability to the BPs *in utero* exposure.

Furthermore, consistent behavioral disturbances were also reported in animal models due to exposure to BPA alternatives during fetal development; in offspring of mice exposed to BPF during gestation, significant increases in anxiety and depressive symptoms were observed, with BPF showing more potent effects than BPA [34]. Similarly, in zebrafish embryos treated with a low dose of BPF and BPA that is 1000-fold lower than the accepted for human daily exposure, the results showed significant increases in neuronal birth (neurogenesis) within the hypothalamus by 240% and 180%, respectively. Increased hypothalamus neurogenesis can lead to different cognitive disorders, such as hyperactivity; indeed, a later development of hyperactive behavior was observed in the zebrafish larvae [35]. In another study, BPS-exposed zebrafish embryos showed a decrease in the locomotor behavior (movement of fish) attributed to the expression of multiple neurodevelopmental genes [36]. Consistently, alterations in the expression of more than 2000 genes, mainly within the amygdala, were observed in the offspring of rats exposed during gestation to a mixture of BPA, BPS, and BPF. The exposed dams showed less potent results than their offspring, suggesting significant time-depending effects as early neurodevelopment is more sensitive to toxicants [37]. Other than suppressing or altering the expression of neurodevelopmental genes, BPA and its alternatives could impact fetal cognitive functions and psychomotor skills through alterations in placental functioning and development. In BPS-exposed placentas, an increase of nearly three-fold in the protein expression of glial cell missing-1 (GCM1) was observed [32]. GCM1 is a transcription factor responsible for controlling the development and function of astrocytes in the central nervous system. Indeed, alterations or abnormalities in the astrocyte were shown to contribute to cognitive impairments related to certain neurological disorders [69,70]. Therefore, increased GCM1 due to BPS exposure could indirectly impact cognitive functions. Moreover, a reduction in dopamine and serotonin content of the BPS-exposed placenta was also reported [31], both of which play roles in the fetal brain structures and functions [51,71]. On the other hand, BPA alternatives may alter the child’s neurodevelopment and motor skills via their roles in interfering with specific hormone activities, including thyroid hormones. BPS exposure at environmentally relevant concentrations was found in multiple studies to alter the gene expression of thyroid hormone receptors [72,73,74]; the maternal thyroid hormones are well-known to play critical roles in the progression of fetal neurodevelopment [75,76].

To state a simple conclusion, BPA alternatives might potentially promote neurodevelopmental disorders despite the lack of epidemiological studies reporting such findings. In a couple of experimental studies, the exposure to BPA alternatives during fetal development showed multiple cognitive disorders, such as anxiety and depressive symptoms, as well as hyperactivity, which was attributed to alterations in gene expression within the hypothalamus and amygdala. In addition, disturbances in the placental content of specific neuronal hormones, such as serotonin and dopamine, were observed. Further research is highly suggested to investigate the possible neurotoxicity of BPA alternatives.

### 3.4. Effects on Bone Development

Prenatal exposure to BPS was linked to multiple abnormalities in the skeletal development of school-aged children [27]; maternal urinary concentrations of BPS in the first trimester were associated with a decrease of 6 points in the children’s bone mineral density at 10 years old. This association showed an age- and trimester-dependent manner; decreased bone mineral density was only observed at 10 years old but not at 6. However, the variability of bone mass outcomes at 6 years old is less than that at 10 years, which explains the obtained results. Further, this association was not linked to either the second or third trimester’s maternal urinary BPS, which may suggest different sensitivity depending on the exposure period. However, considering that fetal skeletal development begins in the first trimester and forward explains the more sensitivity observed. Indicating that *in utero* exposure to BPS during the initiation of fetal skeletal development could lead to persistent effects on the children’s bone development. Nonetheless, it should be considered that the results reported by van Zwol-Janssens et al. [27] were only observed among women who did not take folic acid supplementation. Folic acid plays a role in bone health by promoting the process of DNA methylation, which is a prosses that plays a role in bone health by regulating gene expression patterns involved in bone formation, remodeling, and maintenance [77,78]. Therefore, folate supplementation can be suggested as a potential preventive against the adverse effects of environmental exposures, including BPA and its alternatives. However, further research to establish definitive conclusions is recommended, taking into account that other studies indeed showed potential adverse effects on bone development concerning BPA alternatives; in human osteoblast tissues exposed to different doses of BPF or BPS, the expression of multiple osteogenic markers were observed [79].

Similarly, cell proliferation showed significant dose-dependency inhibition in human osteoblast tissues exposed to BPF or BPS [80]. Osteoblasts are specialized cells responsible for bone formation and are crucial in maintaining bone health and integrity. Therefore, these results suggest that BPF and BPS could potentially threaten bone health. Therefore, further investigations are needed to understand better the specific mechanisms and the potential prenatal consequences of BPA alternatives on fetal bone health.

### 3.5. Effects on Metabolic Parameters

Metabolic disturbances were observed in offspring of different animal models exposed to BPA alternatives during early life development; in male offspring of mice exposed to BPS during gestation and the lactation period, increases in body weight, epididymal white adipose tissue, liver triglyceride, and cholesterol, as well as liver lipid accumulation, were observed. The histopathological examination also showed a significant lipid accumulation in the liver and epididymal white adipose tissues [38]. These results indicate that perinatal exposure to BPS may increase the risk of developing obesity during childhood. Similarly, increased adiposity and liver fat were observed in prenatal BPS-exposed adult male offspring. Notably, these effects were more potent with BPS exposure than with BPA, providing more evidence of the potential higher toxicity of BPA alternatives [39]. Increased body weight with decreased glucose tolerance and insulin sensitivity was also observed in the male offspring of rats exposed to BPS before pregnancy [40]. A significant increase in glucose tolerance and pancreatic β-cell proliferation was also observed in offspring of BPS-exposed mice at low doses [41]. Consistently, in male offspring of mice exposed to BPS during gestation, results showed increased body weight, dyslipidemia, liver triglyceride levels, adipocyte hypertrophy, and hepatic lipid deposition [42]. Disturbances in lipid metabolism were also observed in the offspring of BPS-exposed zebrafish with transgenerational effects. The observed disturbances in the zebrafish dams can also lead to similar disturbances in their offspring [43]. However, it is not precise whether the transgenerational effect can be observed with other effects, as toxicant exposure during fetal development can promote more potent results due to the sensitivity of this period, as mentioned earlier.

The potential mechanisms in which BPA alternatives impact the metabolic system can be linked to various factors; in a cohort study of mother–infant pairs, the maternal BPS urinary concentrations were linked to a decrease of nearly 60% in the mitochondrial DNA copy number (mtDNA, mitochondrial DNA content) of the male infants’ cord blood [28]. Decreased mtDNAcn indicates impaired mitochondrial function, leading to major energy production disturbances, resulting in metabolic dysfunctions. Indeed, studies have demonstrated an association between mtDNAcn in cord blood, insulin levels, and metabolic hormones [81,82]. In another cohort of mother–infant pairs, a decrease of 3.19% in cord blood telomere length was observed in relation to each 1-fold increase in BPS maternal urinary concentrations [29]. Cord blood telomeres are protective structures located at the ends of chromosomes within the cells found in umbilical cord blood; shortened telomeres have been associated with metabolic dysfunctions, such as insulin resistance [83,84,85]. On the contrary, the ability of BPA alternatives to prompt such disturbances in cord blood could be attributed to their role in increasing inflammation and oxidative stress. Both cord blood telomere and mtDNAcn are vulnerable to oxidative damage due to constant exposure to reactive oxygen species [86,87,88]. Therefore, increased inflammations and oxidative stress from BPA alternative exposure could indirectly contribute to metabolic dysfunctions.

In a short conclusion, the progression and development of metabolic disorders in infants and children might be linked to their perinatal exposure to BPA alternatives. Considerable evidence from several experimental studies suggests the potential of BPA alternatives to contribute to abnormalities in metabolic parameters, such as increased lipid accumulation, fatty liver, insulin resistance, and glucose intolerance, leading to an increase in the risk of obesity and type 2 diabetes. The possible mechanism underlying these effects is related to disturbances in cord blood parameters, as well as increased inflammations and oxidative stress as a result of BPA alternative exposure, not to mention that both inflammation and oxidative stress are well-demonstrated in promoting various health issues, including those mentioned earlier [89,90].

## 4. Potential Mechanisms Underlying the Effects: Brief Overview

**Figure 1:**
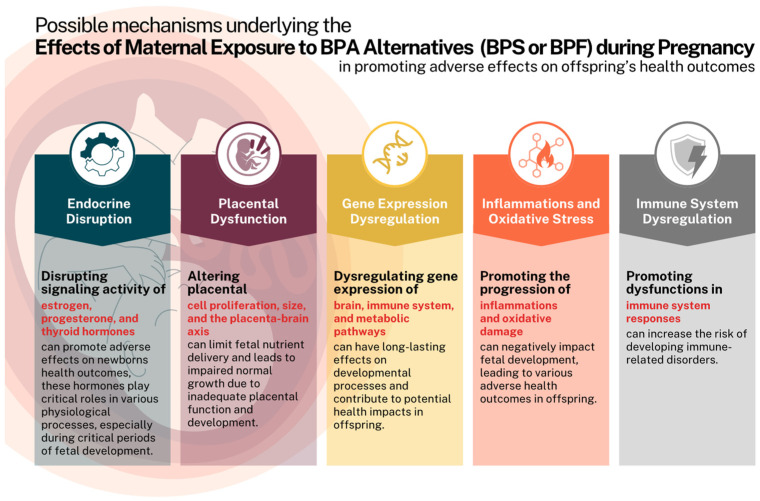
The most reported possible mechanisms of action for BPA alternatives (BPS or BPF) in promoting adverse effects on fetal development. The figure was created by the authors using the Canva website tool.

Some specific mechanisms related to certain health effects were discussed in depth in previous sections; however, the effects of BPA alternatives cannot be solely attributed to a single mechanism. Multiple mechanisms can act in combination or interact with each other, leading to complex and interconnected effects on different biological pathways and systems. As presented in Figure 1, there are multiple possible mechanisms in which prenatal exposure to BPA alternatives contributes to the progression and development of different adverse effects in offspring. One of the main mechanisms is related to the endocrine-disrupting properties of BPA alternatives. These components, when absorbed by the human body at certain concentrations, can interfere with the signaling activity of different hormones, particularly estrogen, progesterone, and thyroid hormone. These hormones are involved in regulating different physiological processes during the critical period of fetal development [33,63,64,65]. Another critical mechanism is the promotion of disturbances in placental functioning and development. The placenta is the most significant tissue related to fetal well-being; therefore, any potential alterations in its integrity can lead to limitations in fetal nutrient delivery and promote fetal growth restrictions. Studies have shown that exposure to BPA alternatives can promote placental cell apoptosis, decrease placental size, and alter the placenta–brain axis [22,23,31]. Moreover, BPA alternatives’ exposure was shown to promote alterations in the gene expression of different tissues and organs during pregnancy, including the brain, immune system, or metabolic system. These alterations might have long-lasting effects on the developmental processes of the offspring and contribute to the progression of different health issues later during childhood development [72,73,74].

Furthermore, induction of inflammations and oxidative damage were also linked to exposure to BPA alternatives. Both of these are well demonstrated in promoting impaired fetal development and contributing to various adverse outcomes in offspring [36,39,45,46,47]. Lastly, modulations in immune system responses have been reported after exposure to BPA alternatives during gestation and lactation periods. Consequently, impaired immunity can increase the risk of different immune-related disorders [44].

## 5. Potential Mechanisms of Sex-Specific Effects

Multiple studies have consistently demonstrated sex-specific effects, indicating varying susceptibility to the adverse effects of BPA alternatives depending on the fetal sex. The precise mechanisms responsible for these findings remain unclear; nonetheless, there are a couple of possible explanations that may offer some understanding. One of these is the difference in the extent of antioxidants between males and females; higher antioxidant levels were found in females to a greater extent than that present in males. In females, the antioxidant properties of estrogen play a significant role in influencing the expression and activity of certain antioxidant enzymes, such as superoxide dismutase and glutathione peroxidase. Another distinct variation between males and females is the placenta dimorphic structure; male and female fetuses exhibit different placental transcriptomic profiles, leading to different responses to environmental toxicants [91]. Studies have shown that the female placenta demonstrates excellent surface differentiation and possesses various survival mechanisms to eliminate adverse events compared to the male placenta [92]. These factors may potentially account for the sex differences observed in fetal sensitivity to the BPA alternatives exposure.

## 6. Strengths and Limitations

There are some strengths and limitation factors that were identified in our work that should be acknowledged to facilitate future research and address potential gaps. One of its primary strengths lies in the inclusion of a large sample size in the majority of the epidemiological studies we reviewed. The large sample size enhances the statistical power and generalizability of the findings as it helps reduce the risk of random variation and increases the confidence in the results. In addition, some of the reported risk factors associated with prenatal exposure to BPA alternatives were derived from longitudinal studies, providing valuable insights into the long-term effects.

However, a couple of limiting factors need to be addressed to provide more comprehensive insights for future investigations. Firstly, many studies were limited to assessing exposure during specific trimesters. To obtain a more comprehensive analysis of potential effects, exposure assessment should be performed across the entire gestational period. This will ensure a more comprehensive understanding of the potential risks. Secondly, several studies relied on a single spot of urine sample for bisphenol assessment. Bisphenols are rapidly metabolized in the human body. Thus, the use of multiple spots of urine samples should be employed to ensure accurate and comprehensive exposure assessment. Third, the eligible epidemiological studies selected did not consider an adjustment for urinary grams of creatinine, which was initially excluded to streamline the comparison process. However, it is crucial to include creatinine adjustment to obtain a more accurate measure of the actual concentration of bisphenols in urine. This adjustment is necessary for a better evaluation and interpretation of bisphenol levels among individuals with varying urine dilution rates, such as variations caused by hydration status. Therefore, this adjustment must be considered in future research to enable standardized measurements and enhance the accuracy of exposure assessments in population studies. Fourth, and lastly, there was a huge geographical bias in the studies we reviewed; a predominance of epidemiological research was conducted in China. This limitation may restrict the generalizability of the findings to a global context, as population characteristics, environmental factors, and exposure levels may differ across different regions worldwide. Therefore, we recommend the urgent need for future investigations to be conducted in other countries to provide a more diverse range of information and enhance the applicability of the findings.

## 7. Conclusions

The currently available findings suggest that exposure to BPS or BPF during early fetal development may contribute to the development of various health issues, such as fetal growth restrictions, neurological disturbances, and metabolic disorders. Maternal exposure to these bisphenols may also shorten the gestational period, increasing the risk of preterm births. BPS and BPF share structural and characteristic similarities with BPA. They have the potential to disrupt endocrine balance, leading to the dysregulation of multiple physiological pathways. Studies have also reported multiple possible mechanisms underlying BPS and BPF potential toxicity on fetal development, including disturbances in placental functioning and development, dysregulation of gene expressions, impairments in immune responses, and inducing chronic inflammations as well as oxidative stress. Further research efforts are strongly recommended to comprehensively understand the potential impacts of BPA alternatives on fetal development and to confirm their toxicity. It should be noted that the majority of the reported epidemiological studies have been conducted in China, highlighting the need for additional studies in other populations worldwide.

## Figures and Tables

**Table 1:** Summary of epidemiological studies reporting associations between maternal urinary concentrations of BPA alternatives (BPS and BPF) and health outcomes in offspring.

Study Design/Location	BPs Exposure Level MUC (µg/L)/DR (%)	Main Outcomes	Reference
Participant Characteristics			
Cohort/China *n* = 1197 mother–newborn pairs (mothers’ age > 18 years; during the late gestation period, 35–47 weeks; singleton pregnancy)	BPS: 0.40 */47.9 BPF: 0.57 */77.1	*BPF in the 3rd TM was associated with*: ↓ birth length by 0.21 cm in female newborns; [95% CI: −0.36, −0.07; *p* < 0.01]. ↑ ponderal index by 0.04 g/cm^3^ × 100; [95% CI: 0.01, 0.08; *p* = 0.02]. *BPS in the 3rd TM was associated with:* ↓ gestational age by 0.20 weeks (linear dose ↓ dependent association in women carrying ↓ female fetuses); [95% CI: −0.37, −0.03; *p* = 0.02].	[15]
Cohort/China *n* = 845 mother–infant pairs (mothers’ median age in years, < 25 (12.4%), 26–35 (83%), and ≥ 36 (4.9%); during full gestation; singleton pregnancy)	*In the 1st, 2nd, and 3rd TMs,* *respectively/average DR:* BPS: 0.3, 0.4, and 0.4/86.8 BPF: 0.6, 0.7, and 0.7/98.3	*BPF in the 1st TM was associated with:* ↓ birth weight (grams); [β: −27; 95% CI: −55, 0; *p* < 0.05]. *BPF in the 3rd TM was associated with:* ↓ ponderal index (kg/m^3^ × 100); [β: −0:17; 95% CI: −0.32, −0.02; *p* < 0.05] with each increase in exposure quartiles. *BPS in the 1st TM was associated with:* ↓ birth weight (grams); [β: −38; 95% CI: −65, −11; *p* < 0.05]. ↓ ponderal index (kg/m^3^ × 100); [β: −0:18; 95% CI: −0.34, −0.02; *p* < 0.05]. *BPS in the 2nd TM was associated with:* ↓ birth weight (grams); [β: −43; 95% CI: −71, −15; *p* < 0.05]. ↓ birth length (cm); [β: −0.12; 95% CI: −0.23, −0.02; *p* < 0.05].	[16]
Cohort/China *n* = 322 mother–newborn pairs (mothers’ age ≥ 18 years; during the late gestation period, ≥35 weeks; singleton pregnancy)	BPS: 0.03/52.5 BPF: 0.08/79.8	*BPS and BPF in the 3rd TM:* No significant associations with fetal growth parameters were observed compared to BPA exposure.	[17]
Cohort/Netherlands *n* = 1379 mother–newborn pairs (mothers’ median age 30.5 years; during full gestation; singleton pregnancy)	*In the 1st, 2nd, and 3rd TMs,* *respectively:* BPS: 0.17, 0.03, and ND/- BPF: 0.13, ND, and 0.13/-	*BPS across all three TMs was associated with:* ↑ fetal head circumference (mm); [difference: 0.18; 95% CI: 0.01, 0.34; *p* < 0.05]. *BPS in the 1st TM was associated with:* ↑ fetal head circumference (mm) in 2nd and 3rd TMs; [difference: 0.15; 95% CI: 0.05, 0.26 and 0.12; 95% CI: 0.02, 0.23, respectively; *p* < 0.02]. ↑ fetal weight (g) in 2nd and 3rd TMs; [difference: 0.12; 95% CI: 0.02, 0.22 and 0.16; 95% CI: 0.06, 0.26, respectively; *p* < 0.02].	[18]
Cohort/China *n* = 2023 mother–infant pairs (mothers’ median age 28.2 years; during full gestation; singleton pregnancy)	BPS: 0.09 **/86.9 BPF: 0.44 **/61.8	*BPF mean serum concentrations were associated with:* ↓ birth weight (grams) in boys; [β: −72.51; 95% CI: −136.59, −8.43; *p* = 0.031]. ↓ ponderal index (kg/m^3^ × 100) with BPF, observed more in boys; [β: −0.71; 95% CI: −1.31, −0.10; *p* = 0.021]. *BPS exposure had no significant associations.*	[19]
Cohort/South Korea *n* = 180 mother–infant pairs (mothers’ median age < 30 years (22.8%) and ≥ 30 years (77.2%); 96% during late gestation period, ≥37 weeks; singleton pregnancy)	BPS: 0.1 */- BPF: 0.2 */-	*BPS exposure was associated with:* ↓ birth weight (grams) for each 10-fold increase in adjusted models ***; [β: −44.2; 95% CI: −92.7, 4.4; *p* = 0.07]. *BPF exposure was associated with:* ↑ birth weight (grams); [β: 125.5; 95% CI: 45.0, 205.9; *p* = 0.003].	[20]
Cohort/China *n* = 289 mother–twin pairs (mothers’ median age 30.06 years; during full gestation; twin pregnancy)	BPS: 0.80/- BPF: 2.52/-	*BPF in the 2nd TM was associated with:* ↑ birth weight (grams); [difference: 72.77; 95% CI: 0.84, 144.7; *p* < 0.05]. *BPS exposure had no significant associations.*	[21]
Case–control/United States *n* = 130 preterm birth cases and 350 random control pregnancy (mothers’ median age 25–35 years; during late gestation ≥ 37 weeks; singleton pregnancy)	*In the 3rd TM:* BPS: -/20	*BPS was associated with:* ↑ odds of overall preterm birth (spontaneous and placental); [OR: 2.05; 95% CI: 1.09, 3.89; *p* = 0.03].	[22]
Cohort/China *n* = 850 mother–infant pairs (mothers’ median age 25–35 years; 97.5% during late pregnancy, ≥ 37 weeks; singleton pregnancy)	*In the 1st, 2nd, and 3rd TMs,* *respectively:* BPS: 0.45, 0.44, and 0.50 */62 – 68 ^	BPS had a nonsignificant association with gestation period or preterm birth (in adjusted models).	[23]
Cohort/China *n* = 2023 mother–infant pairs (mothers’ median age 29 years; during full gestation; singleton pregnancy)	BPS: 0.10/- BPF: 0.60/-	*BPs mixture was associated with:* ↑ preterm birth; [OR: 1.52; 95% CI: 1.04, 2.21; *p* < 0.05]. % of contribution: BPF (43.7%), BPS (29.6%) and BPA (26.8%).	[24]
Cohort/Sweden *n* = 803 mother–child pairs (mothers’ median age, 31.4 years; during full gestation; singleton pregnancy)	BPS: 0.07/- BPF: 0.13/-	*BPF was associated with:* ↓ in full IQ scale, more noted in boys; [β: −2.86; 95% CI: −4.54, −1.18; *p* = 0.001]. *BPS exposure had no significant associations.*	[25]
Cohort/China *n* = 463 mother–child pairs (mothers’ median age 25–34 years; gestational median age, 39 weeks; 87.7% singleton pregnancy)	BPS: 0.37/81–88 ^ BPF: 0.68/98–98.5 ^	*BPS highest exposure level* vs. *lowest was associated with:* ↓ in psychomotor development index; [β: −5.52; 95% CI: −10.06, −0.99; *p* = 0.02] observed more in boys; [β: −7.61; 95% CI: −13.99, −1.24; *p* = 0.02]. *BPF exposure had no significant associations.*	[26]
Cohort/Netherlands *n* = 1362 mother–child pairs (mothers’ median age 30.6 years; during full gestation; singleton pregnancy)	*In 1st, 2nd, and 3rd TMs:* BPS: 0.17, 0.03, and 0.03/- BPF: 0.13, ND, and 0.13/-	*BPS in the 1st TM was associated with:* ↓ bone mineral density at 10 years old; [β: −6.08; 95% CI: −9.97, −2.19; *p* < 0.01].	[27]
Cohort/China *n* = 762 mother–newborn pairs (mothers’ median age 28.6 years; during full gestation; singleton pregnancy)	*In 1st, 2nd, and 3rd TMs:* BPS: 0.32, 0.34, and 0.36/-	*BPS in the 1st TM was associated with:* ↓ mtDNAcn of male newborns by 59%; [95% CI: −75.16, −32.58; *p* < 0.001].	[28]
Cohort/China *n* = 801 mother–infant pairs (mothers’ median age 28.3 years; gestational age < 13 weeks; singleton pregnancy)	BPS: 0.10 */90.9 BPF: 0.46 */65.4	*BPS was associated with:* ↓ cord blood telomere length by 3.19%; [95% CI: −6.08, −0.21; *p* < 0.05].	[29]

Abbreviations. ave: average; BPs: bisphenols; BPS: bisphenol S; BPF: bisphenol F; MUC: median urinary concentrations; DR: detection rate (the percentage of urinary samples containing the tested bisphenol); TM: trimester; mtDNAcn: mitochondrial DNA copy number; (↑): increase; (↓): decrease; OR: odds ratio; HR: hazard ratio; CI: confidence interval; ND: not detected because of over 80% of concentrations were below limit of detection; *: presented as geometric mean (arithmetic average) not median; **: geometric mean of serum samples not urine; ***: models adjusted for different factors, including maternal age, education, smoking and drinking status, body mass index, exercise, infant sex, and gestational weeks; ^: range across pregnancy.

**Table 2:** Summary of experimental studies on animals reporting associations between prenatal or perinatal exposure to BPA alternatives (BPS and BPF) and health outcomes.

Model System	BPs Exposure System	Main Outcomes	Reference
	Dose/Period		
Pregnant sheep	Subcutaneous injections BPA or BPS (0.5 mg kg^−1^ BW)/daily from gestational day 30 to 100	*BPA- and BPS-exposed fetal skeletal muscle showed:* ↑ muscle fiber hypertrophy ↑ expression of myogenic regulatory factors	[30]
Mouse dams	Oral administration to dams before pregnancy BPA or BPS (200 μg kg^−1^ BW)/daily for 2 weeks and after breeding until embryonic day 12.5	*Both BPA- and BPS-exposed placentas showed:* ↓ serotonin ↑ dopamine	[31]
Pregnant sheep	Injection to pregnant sheep BPS or BPA (0.5 mg kg^−1^ BW, internal fetal doses ~2.6 ng mL^−1^ of BPA and ~7.7 ng mL^−1^ of BPS)/daily from gestational day 30 to 100	*BPS-exposed placentas showed:* ↓ E-cadherin protein expression by 50%. ↓ binucleate cells by ~20%. ↑ glial cell missing-1 protein expression by ~3 folds	[32]
Pregnant rats	Oral administration to dams during pregnancy BPA, BPS, and BPF (1 or 5 mg kg^−1^ BW)/daily from gestational days 6 to 21	*BPS- and BPF-exposed dams showed:* ↑ spontaneous abortions (over 80% of dams, BPF only) ↓ number of corpora lutea	[33]
Pregnant mice	Oral administration to dams during pregnancy BPA or BPF (0 or 10 mg kg^−1^ BW)/daily from gestational day 11.5 to 18.5	*Prenatal BPF-exposed offspring showed:* ↑ anxiety and depressive state	[34]
Zebrafish embryos	Exposed in a test solution BPA or BPS (0.0068 μM)/daily until day 5 postfertilization	*BPA and BPS exposure showed:* ↑ neurogenesis within the hypothalamus by 180% for BPA and 240% for BPS (resulted in later hyperactive behaviors in the zebrafish larvae)	[35]
Zebrafish embryos	Exposed in a test solution BPS (0, 0.03, 0.3 and 3.0 mg L)/daily until day 6 postfertilization	*BPS exposure showed:* ↓ locomotor behavior ↑ oxidative stress ∆ neurodevelopment genes expression	[36]
Pregnant rats	Oral administration to dams Mixture of BPA, BPS, and BPF (150 µg kg^−1^ BW)/daily from gestational day 8 to day of birth	*Prenatal bisphenols-exposed offspring showed:* ∆ expression of over 2000 genes (amygdala was the most affected region). ↑ genes expression in the hypothalamus	[37]
Pregnant mice	Oral administration to dams during pregnancy BPS (100 μg kg^−1^ BW)/daily from gestational day 7 to postnatal day 21	*Perinatal BPS-exposed male offspring showed:* ↑ body weight ↑ epididymal white adipose tissue ↑ liver triglyceride and cholesterol ↑ liver lipid accumulation	[38]
Pregnant rats	Oral administration to dams BPA or BPS (0.0, 0.4, 4.0 μg kg^−1^ BW)/daily from gestational day 4 to 21	*Prenatal BPS-exposed male offspring showed:* ∆ feed efficiency ∆ leptin signaling ↑ adipocyte hypertrophy ↑ fatty liver ↑ adipose tissue inflammation ↑ oxidative stress in adipose tissue	[39]
Adult rats	Administration through dams’ drinking water BPS (4 and 40 μg kg^−1^ BW)/daily for 2 months before mating	*Prenatal BPS-exposed male offspring showed (lower dose):* ↑ body weight ↓ glucose tolerance ↓ insulin sensitivity	[40]
Pregnant mice	Oral administration to dams during pregnancy and lactation BPS (0.05 or 20 mg kg^−1^ BW)/daily from gestational day 6 to postnatal day 21	*Perinatal BPF-exposed offspring showed:* ↓ body weight ↑ glucose tolerance ↑ pancreatic β-cell proliferation	[41]
Pregnant mice	Oral administration to dams BPS (50 μg kg^−1^ BW)/daily during gestation	*Prenatal BPS-exposed male offspring showed:* ↑ body weight ∆ lipid metabolism ↑ liver triglyceride ↑ adipocyte hypertrophy ↑ hepatic lipid deposition	[42]
Zebrafish	Exposed in a test solution BPS (1, 10, 100, and 1000 mg L^−1^)/daily for 120 days	*Prenatal BPS-exposed offspring showed:* ∆ lipid metabolism	[43]
Mice dams	Dermal exposure to dams BPA, BPS, or BPF (5 or 50 μg kg^−1^ BW)/daily from gestation day 15 to postnatal day 21	*Perinatal BPF-exposed offspring showed:* ∆ immune responses	[44]
Pregnant mice	Exposed to drinking water from dams BPS (1.5 µg kg^−1^ BW)/daily from gestational day 0 until weaning of offspring	*Perinatal BPS-exposed offspring showed:* ↑ inflammatory response (observed in F1 and F2)	[45]
Lactating rats	Oral administration to dams BPF (0.0365 and 3.65 mg kg^−1^ BW)/daily	*Perinatal BPF-exposed female and male offspring* *showed:* ↑ liver inflammation	[46]
Lactating rats	Oral administration to dams BPF (0.0365 and 3.65 mg kg^−1^ BW)/daily until postnatal day 6	*Perinatal BPF-exposed female and male offspring* *showed:* ↑ lipid peroxidation ↑ oxidative stress	[47]

Abbreviations. BPs: bisphenols; BPA: bisphenol A; BPS: bisphenol S; BPF: bisphenol F; BW: body weight; (↑): increase; (↓): decrease; (∆): alteration/changes.

## Data Availability

Data are contained within the article.
